# Characterization of an advanced viable bone allograft with preserved native bone-forming cells

**DOI:** 10.1007/s10561-022-10044-2

**Published:** 2022-11-25

**Authors:** Elena Gianulis, Bradley Wetzell, Danielle Scheunemann, Patrick Gazzolo, Payal Sohoni, Mark A. Moore, Jingsong Chen

**Affiliations:** 1 Global Scientific Affairs and Clinical Engagement, LifeNet Health®, 1864 Concert Dr., Virginia Beach, VA 23453 USA; 2 Institute of Regenerative Medicine, LifeNet Health®, Virginia Beach, VA USA; 3 Global Spine and General Orthopedics, LifeNet Health®, Virginia Beach, VA USA; 4 Global Trauma and CMF, LifeNet Health®, Virginia Beach, VA USA

**Keywords:** Bone graft, Bone regeneration, Bone void filler, Cellular bone allograft, Viable bone allograft, ViviGen

## Abstract

Bone grafts are widely used to successfully restore structure and function to patients with a broad range of musculoskeletal ailments and bone defects. Autogenous bone grafts are historically preferred because they theoretically contain the three essential components of bone healing (ie, osteoconductivity, osteoinductivity, and osteogenicity), but they have inherent limitations. Allograft bone derived from deceased human donors is one alternative that is also capable of providing both an osteoconductive scaffold and osteoinductive potential but, until recently, lacked the osteogenic component of bone healing. Relatively new, cellular bone allografts (CBAs) were designed to address this need by preserving viable cells. Although most commercially-available CBAs feature mesenchymal stem cells (MSCs), osteogenic differentiation is time-consuming and complex. A more advanced graft, a viable bone allograft (VBA), was thus developed to preserve lineage-committed bone-forming cells, which may be more suitable than MSCs to promote bone fusion. The purpose of this paper was to present the results of preclinical research characterizing VBA. Through a comprehensive series of in vitro and in vivo assays, the present results demonstrate that VBA in its final form is capable of providing all three essential bone remodeling properties and contains viable lineage-committed bone-forming cells, which do not elicit an immune response. The results are discussed in the context of clinical evidence published to date that further supports VBA as a potential alternative to autograft without the associated drawbacks.

## Introduction

Bone grafts have been used to successfully restore structure and function to patients with a broad range of musculoskeletal ailments and bone defects for more than a century (Albee 1915; de Boer 1988; James 1974). Bone grafting continues to be commonly employed by surgeons across a variety of disciplines to treat bone defects throughout the body (Khan et al 2005) with over a half-million procedures performed annually in the US (Greenwald et al 2001). Autogenous bone grafts are historically preferred because they theoretically contain the three essential components of bone healing: an osteoconductive scaffold to support bone formation, osteoinductive molecular signals to promote it, and osteogenic cells to produce the new bone (Khan et al 2005). However, the availability of autograft bone is inherently limited and the additional surgical procedure increases operative time and cost, blood loss, and postoperative pain (Younger and Chapman 1989). Further, autograft quality is potentially constrained by patient age, comorbidities, lifestyle risks, and intraoperative processing methods (Khan et al 2005). As such, alternative graft materials have emerged with the goal of replicating the benefits of autograft bone while mitigating its drawbacks.

Allograft bone derived from deceased human donors is one such alternative, coming in a variety of forms, shapes, and sizes for a wide array of surgical applications. Mineralized grafts, such as cancellous cubes or ground cortical particulate, are among the most basic bone allografts and provide an excellent osteoconductive scaffold for new bone growth and incorporation into the host (Khan et al 2005), yet they lack osteoinductive and osteogenic components. Demineralized bone matrices (DBMs), on the other hand, are processed to partially remove the mineral matrix, while retaining the collagen matrix, thus exposing native growth factors in the bone, such as bone morphogenetic proteins (BMPs) and angiogenic factors (Hankenson et al 2011; Zhang et al 1997). Thus, DBMs provide both osteoinductive potential and an osteoconductive scaffold (Turonis et al 2006); however, they still lack an osteogenic component for bone remodeling.

Cellular bone allografts (CBAs) are a relatively new class of graft options that were designed to address this need by preserving native viable cells with osteogenic potential within an osteoconductive bone matrix. Demineralized bone is also typically included to enhance the graft’s osteoinductivity and provide an additional osteoconductive scaffold. Thus, CBAs can theoretically provide all three necessary components of bone healing, similar to autogenous bone, but without the aforementioned drawbacks. Most commercially-available CBAs feature mesenchymal stem cells (MSCs) as their osteogenic component. Although MSCs have the potential to develop into bone-forming cells, this process is time consuming, requiring induction and osteogenic differentiation (Birmingham et al 2012). Osteogenic-lineage differentiation relies upon molecular signals in the local environment, which may vary from patient-to-patient and thus may not provide the ideal conditions for development into bone-forming cells (Tortelli et al 2010). This means that MSCs could also develop into unwanted cell types, such as muscle, nerve, or fat cells, which has the potential to delay or inhibit bone formation and fusion.

A more advanced bone graft, a viable bone allograft (VBA; ViviGen^®^ and ViviGen Formable^®^; LifeNet Health^®^, Virginia Beach VA), was thus developed to remove these uncertainties by uniquely preserving native lineage-committed bone-forming cells within a corticocancellous matrix combined with demineralized bone. Evidence suggests that these types of lineage-committed cells are more suitable than MSCs to promote bone fusion (Birmingham et al 2012; Ghanaati et al 2011; Tortelli et al 2010). Since its introduction, VBA has been widely used across a variety of disciplines, including spine, trauma, foot and ankle, craniomaxillofacial, and oral surgery. Here, we present the results of preclinical research for the characterization of VBA and discuss them within the context of subsequent published clinical evidence.

## Methods

### VBA preparation

**Fig. 1:**
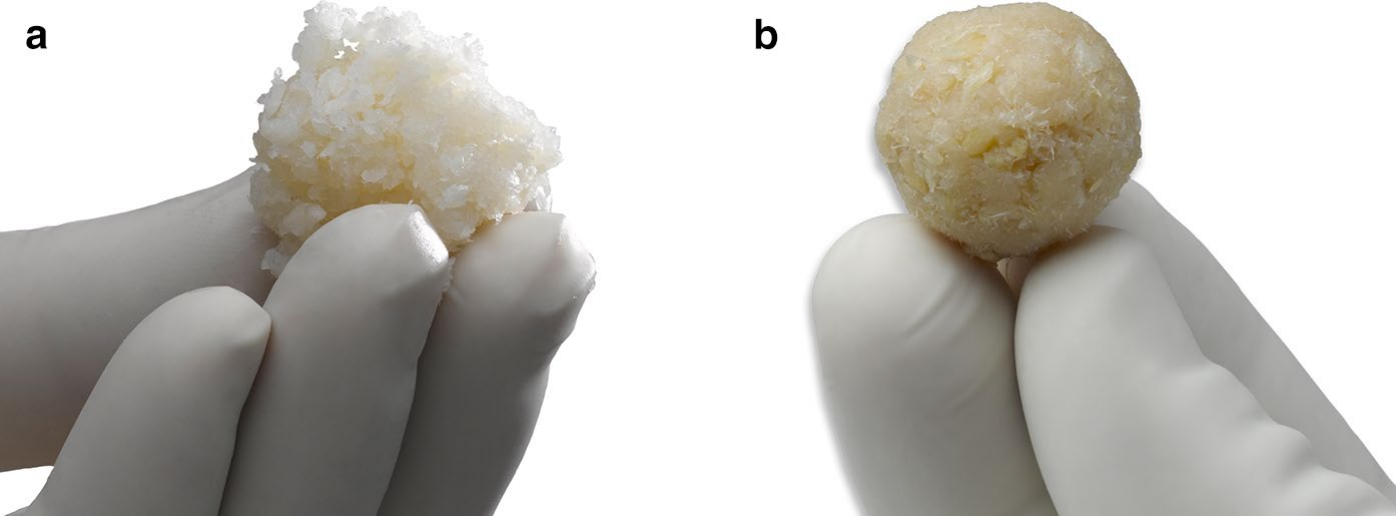
Representative photographs of VBA in its final form with either a **a** particulate or **b** fiber demineralized component

VBA is comprised of two main components, each derived from the same human donor: chips prepared from the corticocancellous portion of bones known to contain bone cells and DBM prepared from the cortical portion of long bones. After aseptic recovery and debridement, the corticocancellous pieces are ground into chips, and the cortical bone is either ground into particulate or prepared as long fibers using a proprietary computer-numerical-controlled (CNC) milling method, followed by cleansing of each component. The cortical particulate or fibers are then demineralized using proprietary procedures. In VBA’s final form, both the viable chips and demineralized component (as either particulate or fibers) are combined (Fig. 1a,b, respectively) with cryopreservation medium into a proprietary thin-walled package and cryopreserved in liquid nitrogen. Each lot of VBA corresponds to only one donor. The characterization assays outlined in the following sections were conducted on individual lots derived from research-consented donors. Unless noted otherwise, VBA was prepared in its final form, cryopreserved, then thawed according to the published instructions for use (LifeNet Health 2019).

### Preliminary characterization

#### *Cell viability*

To initially establish whether VBA contains viable cells following standard proprietary processing, cryopreservation, and thawing, lactate dehydrogenase (LDH) activity staining was performed on samples of VBA in its final form. As originally reported by Wong and colleagues (1982), LDH activity in osteocytes has long been utilized as an indicator of bone tissue viability, as it remains stable for up to 36 h after cell death. For the present assay, newer refinements described by Jähn and Stoddart (2011) were applied to maximize penetration of the metabolically-active reagents to the central cell populations within the three-dimensional bone tissue samples and to permit better visualization of individual viable cells within them. Briefly, the assay supplies nitroblue tetrazolium salt as a third substrate alongside lactate and oxidized nicotinamide adenine dinucleotide (NAD^+^), which is taken up by viable cells and converted by the LDH enzyme to a water-insoluble formazan, resulting in dark violet-stained osteocytes. Stained samples were then fixed in 10% neutral-buffered formalin (NBF), decalcified with a 0.35 M EDTA solution (pH 7.2), and placed on glass slides without mounting for brightfield visualization.

To next verify that these cells remain viable and capable of proliferation, VBA samples were plated in 6-well tissue culture treated plates at approximately 1 cc/well following standard proprietary processing, cryopreservation, and thawing. The samples were then covered in growth media composed of Dulbecco’s Modified Eagle’s Medium (DMEM) supplemented with 10% fetal bovine serum (FBS) and 1% antibiotic/antimycotic (ABAM; all from Invitrogen/Thermo Fisher Scientific, Waltham MA). Following a 4- to 6-week incubation at 37 °C and 5% CO_2_ with regular monitoring and media changes, the wells were observed via brightfield microscopy for evidence of cell outgrowth from the bone chips.

#### *Osteoblast-related gene and protein expression*

To determine whether the cells preserved within VBA express genes associated with osteoblasts, quantitative reverse-transcription polymerase chain reaction (qRT-PCR) analyses were performed to examine the RNA expression in cell lines isolated from VBA (VBA bone cells; V-BC) for bone morphogenetic protein-2 (BMP-2) and the osteoblast-associated signaling molecules osteopontin (also known as bone sialoprotein) and osteocalcin (Vaananen et al 2000; Zoch et al 2016). For V-BC cells, VBA samples from six separate lots were plated as described in the preceding methods for cell outgrowth. Once the observed outgrowths became confluent, the cells were isolated, passaged, and frozen for storage until analysis. Commercially-obtained normal human osteoblast cells (NHOst; Lonza Biotech, Morristown NJ) were used as a positive control and human bone marrow mesenchymal stem cells (hMSC; Lonza Biotech, Morristown NJ) were used as a comparator.

For analysis, V-BC, NHOst, and hMSC were thawed, plated at 1 × 10^5^ cells/cm^2^ in 6-well plates, and propagated in growth media, as described, until the cells were approximately 75% to 85% confluent. Total RNA was extracted using TRIzol™ reagent (Invitrogen/Thermo Fisher Scientific, Waltham MA), according to the manufacturer’s protocol, and RNA purity and concentration were analyzed with a NanoDrop^®^ spectrophotometer (NanoDrop Technologies, Wilmington DE). Reverse transcription was carried out using the RT^2^ cDNA synthesis kit and protocol (SA Biosciences/QIAGEN, Germantown MD), and expression levels were measured using TaqMan^®^ Master Mix and probes (Applied Biosystems®/Thermo Fisher Scientific, Waltham MA) according to the manufacturer’s protocol. After normalizing the Cycle Threshold (Ct) values from each cell line to their respective internal controls (GAPDH), V-BC and hMSC expression levels were set relative to those of NHOst using the 2^−∆∆Ct^ method.

To establish whether V-BC express proteins associated with osteoblasts, immunocytochemistry (ICC) was performed on cells cultured from VBA as described in the preceding methods for qRT-PCR. V-BC cells were seeded onto chamber slides, fixed in 10% NFB, and incubated with anti-human osteocalcin purified mouse monoclonal IgG primary antibody (R&D Systems, Minneapolis MN) and Northern Lights anti-mouse IgG-NL557 (R&D Systems) as a secondary. The cells were then counterstained with hematoxylin to identify cell nuclei and mounted for brightfield visualization of osteocalcin expression.

To next confirm protein expression of in-situ cells within the bone matrix before and after VBA processing, immunohistochemistry (IHC) was performed to assess the presence of osteocalcin. The preprocessing sample consisted of bone matrix collected immediately after grinding without further processing. The postprocessing sample was subsequently collected as fully processed VBA from the same lot. Each sample was immediately fixed in 10% NFB and 1 cc was decalcified using a 0.35 M EDTA solution (pH 7.2), embedded, sectioned, and incubated with anti-human osteocalcin mouse monoclonal IgG primary antibody (Abcam, Waltham MA) and EnVision+ mouse HRP polymer (Dako Agilent, Santa Clara CA) as a conjugated secondary. Hematoxylin was used as a counterstain to identify cell nuclei. Positively-stained cells were examined via brightfield microscopy to detect cells expressing osteocalcin at each processing timepoint.

### Properties of bone formation

#### *Osteoconductivity*

To determine whether seeded cells were able to attach and spread along VBA, scanning electron microscopy (SEM) was used to qualitatively evaluate the attachment and morphology of bone marrow-derived mesenchymal stem cells (bmMSCs) and V-BC. bmMSCs were isolated from bone marrow aspirate collected at the time of recovery from the vertebrae of a research-consented donor and characterized by flow cytometry for MSC-specific surface markers, as well as chondrogenic, adipogenic, and osteogenic differentiation assays (data not shown). The density of seeded cells was 62,500 cells per 60.5 (± 1) mg of demineralized bone. Cells were cultured in their respective growth media for up to 7 days. After 1 h, 1 day, and 7 days in culture, the media were removed, and samples were fixed with 2.5% glutaraldehyde in cacodylate buffer and stored at 4 °C until imaging (University of Virginia Advanced Microscopy Facility, Charlottesville VA). Prior to imaging, samples were washed in 0.1 M cacodylate buffer, fixed in 1% osmium tetroxide, dehydrated in a series of alcohol solutions from 30 to 100%, dried using hexamethyldisilane, and finally sputter coated in gold palladium for 200 s at 60 mA.

To evaluate whether the demineralized component of VBA provides a biocompatible environment, an alamarBlue^®^ assay (Bio-Rad™, Hercules CA) was used to measure the metabolic activity of seeded cells over time. V-BC and bmMSCs from six lots were seeded on the demineralized component of VBA at 62,500 cells/well. After 1, 4, and 7 days, the media were removed and 1 mL of 10% alamarBlue was added to each well and incubated for 2 h at 37 °C. The solution was collected and 50 µL was transferred to a black-walled, clear-bottomed, 96-well plate. The fluorescence of the samples was read at an excitation wavelength of 544 nm and emission wavelength of 592 nm. For each timepoint, results of three triplicates were reported as mean (standard error; SE) and statistical comparisons between timepoints were performed using a one-way ANOVA with Tukey’s post-hoc tests, where *p* < 0.05 was considered statistically significant.

As an additional test of biocompatibility, a PicoGreen DNA quantification assay (Quant-iT™ PicoGreen™ dsDNA Assay Kit; Molecular Probes, Inc., Eugene OR) was used to measure the total DNA and number of cells attached to the demineralized component of VBA. On Day 7, cells from the previous alamarBlue assays were collected for DNA quantification. After removing the media, the cells were washed three times with phosphate-buffered saline to completely remove any residual alamarBlue solution. Next, cells were digested by adding 1 mL of Tris–EDTA (TE) buffer with 30 µL of Proteinase K to each well. After an incubation of up to 3.5 h at 65 °C with agitation, 100 µL of 1 × PicoGreen was added to 100 µL of sample digest in black-walled, clear-bottomed, 96-well plates and measured at an excitation wavelength of 485 nm and emission wavelength of 538 nm. Samples were measured in triplicate and DNA was quantified using a standard curve calculated from serial dilutions of calf thymus DNA (Sigma Aldrich^®^, St. Louis MO). To correlate DNA mass to cell number, DNA quantification of the initial cell suspension of 62,500 cells was measured on Day 0, to quantify the amount of DNA per cell, and then again on Day 1 from the media that was removed to quantify DNA from cells which did not attach. The difference between the DNA from the initial cell suspension and that from the media supernatant taken on Day 1 corresponds to the number of cells that attached to the demineralized component. These values were subsequently compared to the DNA quantified on Day 7, providing a measure of the level of cell proliferation on the demineralized component. Values for each timepoint were reported as mean (SE) and statistical comparisons between timepoints were made using a one-way ANOVA with Tukey’s post-hoc tests, where *p* < 0.05 was considered statistically significant.

#### *Osteoinductivity*

To evaluate the presence of osteoinductive growth factors BMP-2 and BMP-7 in vitro, the demineralized component of VBA derived from six lots was analyzed using an enzyme-linked immunosorbent assay (ELISA; Quantikine^®^; R&D Systems, Minneapolis MN). Demineralized bone samples were digested with 1 mg/mL collagenase enzymatic solution (Gibco™, Waltham MA). After an 18-h incubation with agitation followed by centrifugation, the digested samples were then treated with 4 M Guanidine Hydrochloride (GuHCL) at a ratio of 0.2 g of tissue to 1 mL of 4 M GuHCL. Following a 20-h incubation with agitation, the samples were centrifuged, and the supernatant was collected. The sample pellet was resuspended in 5 mL of DMEM and vortexed at maximum speed for 30 s followed by centrifugation. The supernatant was collected and mixed with the previously collected supernatant. The protein solutions were analyzed in triplicate per the manufacturer’s instructions using the Thermo Scientific Multiskan™ GO spectrophotometer at 450 nm. The measured BMP content was calculated according to the standard curves plotted on a logarithmic scale, which were calculated from serial dilutions of either BMP-2 or BMP-7 protein concentrate (R&D Systems, Minneapolis MN). The concentrations were averaged across all six lots and reported in pg protein/mL of protein elution.

The presence of angiogenic growth factors, vascular endothelial growth factor (VEGF) and angiogenin, within the demineralized component of VBA derived from six lots was quantified using the MAGPIX^®^ Protein Multiplexing System (Lumenix^®^, Austin TX). Samples were digested as previously described for the ELISA assay. The resulting solutions were analyzed in duplicate following the manufacturer’s instructions, whereby 50 µL of standard or sample was added to each well of the provided microplate and treated with assay diluent. Magnetic microparticles pre-coated with analyte-specific antibodies were added to each well and allowed to incubate for 2 h. Following wash steps, the reagents were replaced with a biotin-antibody cocktail and then Streptavidin-PE solution. The Luminex MAGPIX analyzer was used to measure the excitation levels (fluorescence intensity) captured by two light-emitting diodes. Concentrations of each protein were calculated according to the standard curve produced by a five-parameter logistic fit model and were reported in pg protein/mL of protein elution.

The osteoinductive potential of VBA and capability to form new bone in vivo was determined using an athymic mouse intermuscular pouch model at the NAMSA facility per IACUC protocol and test code (T0766-002/s). The demineralized component from six lots (20 to 25 g per replicate; 4 replicates for each sample) were rehydrated with saline and loaded into a 1 cc syringe for delivery. The samples were implanted inter-muscularly between the biceps femoris and superficial gluteal muscle. The implants were recovered 5 weeks post-implantation and fixed in 10% NBF, decalcified, and embedded in paraffin. Sections were stained with hematoxylin and eosin (H&E) for histological assessment.

#### *Osteogenicity*

To demonstrate the mineralization capability of V-BC, Alizarin Red S (Sigma Aldrich^®^; St. Louis MO) was used to evaluate calcium deposition. V-BC isolated from VBA derived from three lots or hMSCs (Lonza Biotech, Morristown NJ) were seeded in 24-well plates at a density of 1 × 10^5^ cells per cm^2^. Cells were cultured for 7, 14, and 21 days in either growth media (GM) or osteogenic differentiation media (OM; DMEM high glucose, 10% FBS, 50 µM ascobate-2-phospate, 10 mM β-glycerophosphate and 1% antibiotic/antimycotic). At each time point, wells were washed with dH_2_O and fixed with 10% phosphate buffered formalin. Following another wash with dH_2_O, the wells were stained using 2% Alizarin Red S solution (pH 4.1 to 4.3) and evaluated with a phase contrast microscope at 10 × magnification.

### Potential immunogenicity

To evaluate the immunogenic potential of VBA, histology was performed as previously described in “Preliminary Characterization” to assess the presence of bone marrow cells and the cell surface antigen major histocompatibility class II (MHCII) in bone matrix samples before and after processing into VBA. Briefly, both a preprocessing and a postprocessing sample were immediately fixed in 10% NFB, decalcified, embedded, and sectioned. Sections were initially stained for CD45, a marker of hematopoietic cells, or CD166, a marker for MSCs. As follow-up to the immunogenicity assay (described next), additional sections from each processing timepoint were stained for MHCII surface receptors, a marker for antigen presenting cells. For all sections, hematoxylin was used as a counterstain to identify cell nuclei. Positively-stained cells were observed via brightfield microscopy to detect cells expressing each marker at each processing timepoint.

To assess the potential immunogenicity of VBA, a mixed lymphocyte reaction (MLR) assay was performed using V-BC. The MLR assay is a test recommended by the US Food and Drug Administration (FDA) to measure the functional immune response mediated by T-cells against foreign antigens (CBER 1999). In the MLR assay, cells from a donor, acting as a stimulatory cell population, are mixed with a responding human leukocyte antigen (HLA)-mismatched lymphocyte population. If the stimulatory cells are immunogenic, the lymphocytes respond by proliferating (Muul et al 2011). This increase in lymphocyte proliferation is detected by the incorporation of the nucleotide analog bromodeoxyuridine (BrdU) into the DNA of actively dividing cells and subsequently measured quantitatively using an ELISA assay.

V-BC were isolated from VBA samples derived from three separate lots as previously described in “Preliminary Characterization”. However, a portion of one VBA sample underwent proprietary processing but was plated prior to cryopreservation to serve as a “pre-cryopreservation” sample. HLA-matched lymphocytes (LC) were isolated from lymph nodes recovered from the same donors as those for V-BC cells to serve as the positive control for an antigen-induced lymphocyte immune response. Recovered lymph nodes were minced, and the HLA-matched LCs rinsed from the tissue were collected, cultured in non-adherent conditions, and frozen for storage until analysis. Commercially-obtained peripheral blood mononuclear cells (PBMCs; Hemacare^®^, Los Angeles CA) were used as the HLA-mismatched responding cell population.

To perform the MLR assay, V-BC were thawed and allowed to attach at a density of 1 × 10^4^ cells/well in a 96-well plate overnight. The next day, LCs were thawed and plated at 1 × 10^5^ or 2 × 10^5^ cells/well in a separate 96-well plate. Both the V-BC and LCs were then treated with 50 µg/mL mitomycin C (mitoC) for 30 min to inhibit further cell proliferation. Therefore, any cell proliferation detected in subsequent steps would be from the responding cell population (PBMCs) and not the stimulating cells (V-BC or LCs). PBMCs were then thawed and added to mitoC-treated V-BC cells at a ratio of 40 to 1, as the test samples, and to mitoC- treated LCs at a ratio of 4 to 1, as the positive control samples. PBMCs cultured alone at a density of 4 × 10^5^ cells/well served as the negative control. After 3 days of culture, BrdU (10 µM final concentration) was added to each well and allowed to incubate at 37 °C overnight. The next day, the media were removed, and 200 µL denaturation solution was added to each well. After a 30-min incubation at room temperature, 100 µL anti-BrdU antibody was added to each well and incubated for 90 min at room temperature. The antibody solution was removed, 100 µL/well of substrate solution was added, and the amount of incorporated BrdU was quantified by ELISA according to the manufacturer’s protocol (Cell Proliferation ELISA, BrdU; Roche, Indianapolis IN). Sample absorbance levels were taken at 450 nm and reported as mean  (standard deviation; SD) proportion of total BrdU incorporated. Statistical comparisons versus the PBMC-only control were performed using a Student’s *t*-test with *p* < 0.05 considered statistically significant.

## Results

### Preliminary characterization

#### *VBA contains viable cells*

**Fig. 2:**
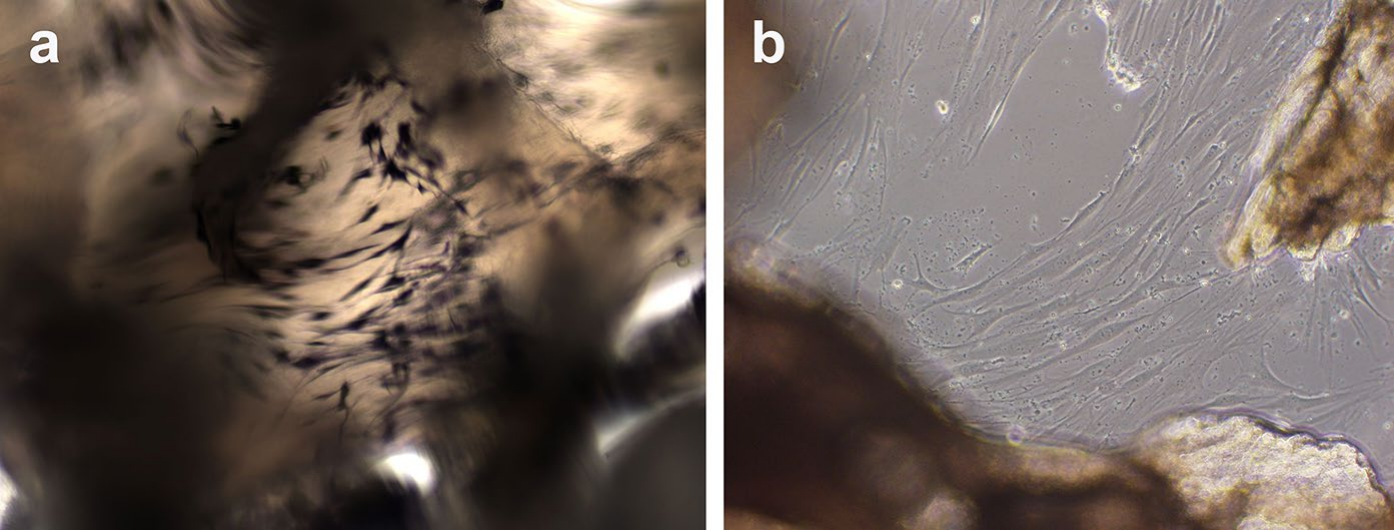
Representative micrographs (10 × magnification) showing **a** dark violet formazan-stained viable osteocytes in a three-dimensional sample of VBA following the LDH activity assay. **b** Following 4 to 6 weeks in growth medium, cells were observed growing from the bone chips into the medium, indicating that they remained generally active and could proliferate

LDH activity staining was used to initially establish that the cells preserved within the VBA bone matrix were viable. Staining indicated that the cells took up the metabolically-active reagents and converted them to formazan, resulting in clearly-defined viable cells embedded within the three-dimensional bone tissue (dark violet stains, Fig. 2a). To next confirm that these cells were able to proliferate, VBA bone chips were plated and incubated in growth medium. Following 4 to 6 weeks, cells were observed growing from the bone chips into the medium (Fig. 2b), indicating that the cells preserved within VBA remained viable and were able to proliferate.

#### *V-BC express osteoblast-related genes and proteins*

**Fig. 3:**
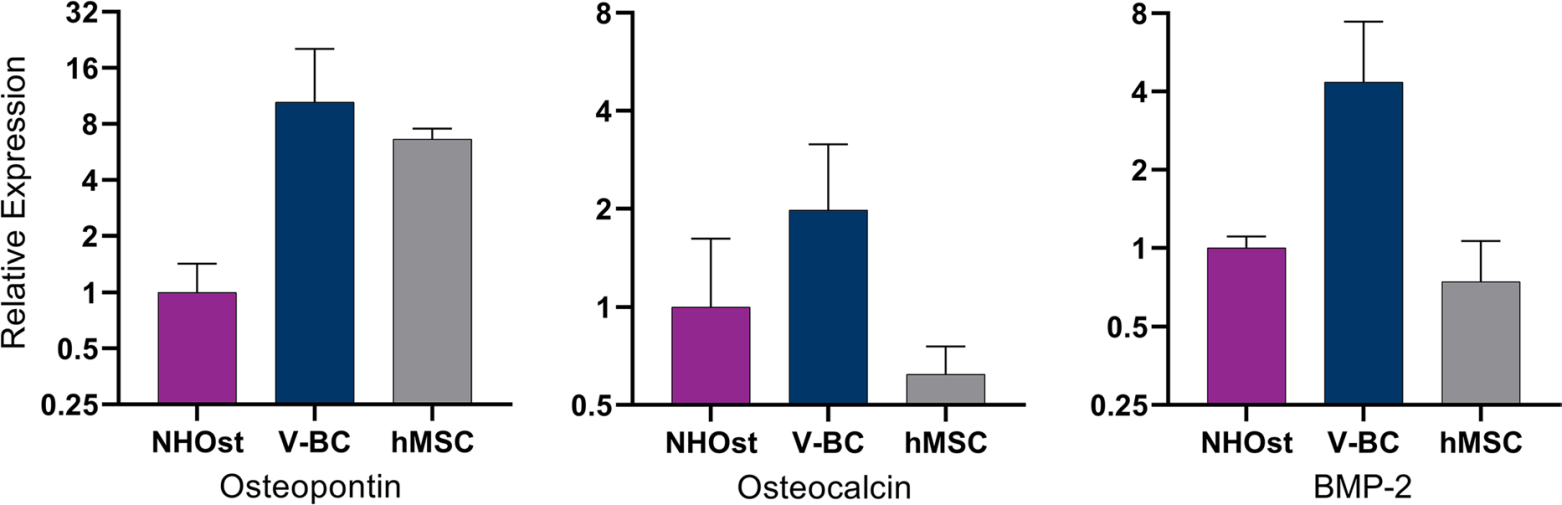
Results of qRT-PCR analyses of osteoblast-related gene expression in cells derived from VBA (V-BC; N = 6) and human mesenchymal stem cells (hMSC; N = 3 replicates), relative to normal human osteoblast controls (NHOst; N = 3 replicates). V-BC expressed genes for osteopontin, osteocalcin, and BMP-2, a putative expression profile suggesting lineage-committed differentiation into osteoblasts

To characterize the viable cells preserved within VBA, RNA expression typically associated with osteoblasts was assessed via qRT-PCR (Fig. 3). V-BC expressed genes for osteopontin, osteocalcin, and BMP-2, which is an expression profile suggesting lineage-committed osteoblasts (Vaananen et al 2000; Zoch et al 2016).

**Fig. 4:**
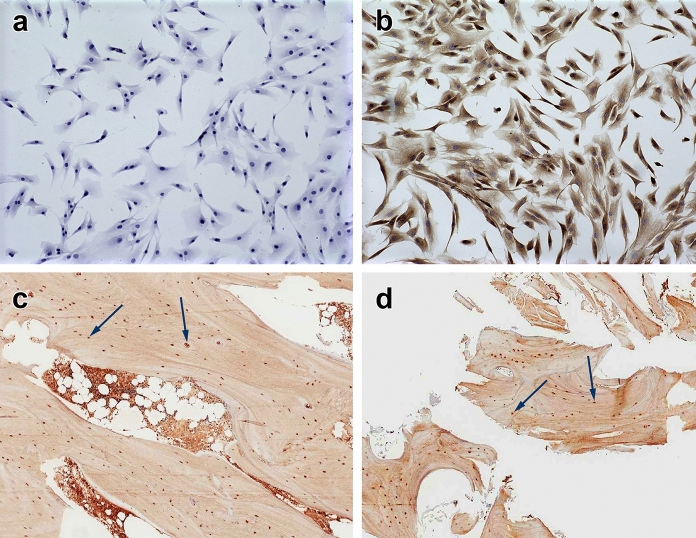
Representative micrographs of ICC and ICH staining for osteocalcin. For ICC (10 × magnification), **a** cells counterstained with hematoxylin (without osteocalcin antibody) showed presence of V-BC cells cultured from VBA, while **b** test samples stained positive for the putative osteoblast marker. IHC staining for osteocalcin (20 × magnification, darker red stain, denoted by arrows) in bone matrix **c** before and **d** after proprietary processing, cryopreservation, and thawing of VBA, demonstrating retention of this putative osteoblast marker in VBA’s final form

To further characterize the cells preserved within VBA, osteocalcin protein expression was assessed in V-BC using ICC, and in situ using IHC on bone matrix before and after VBA processing (Fig. 4). For ICC, cells counterstained with hematoxylin (without osteocalcin antibody) showed the presence of V-BC cultured from VBA (Fig. 4a), while test samples stained positive for the osteoblast marker osteocalcin (Fig. 4b). For IHC, osteocalcin-positive cells were visible both before (Fig. 4c) and after processing of VBA (Fig. 4d), thus demonstrating retention of this putative osteoblast marker in VBA’s final form and providing support for the lineage-committed fate of the cells preserved within VBA.

Properties of bone formation

### *Osteoconductivity: VBA supports cell attachment, spreading, and proliferation*

**Fig. 5:**
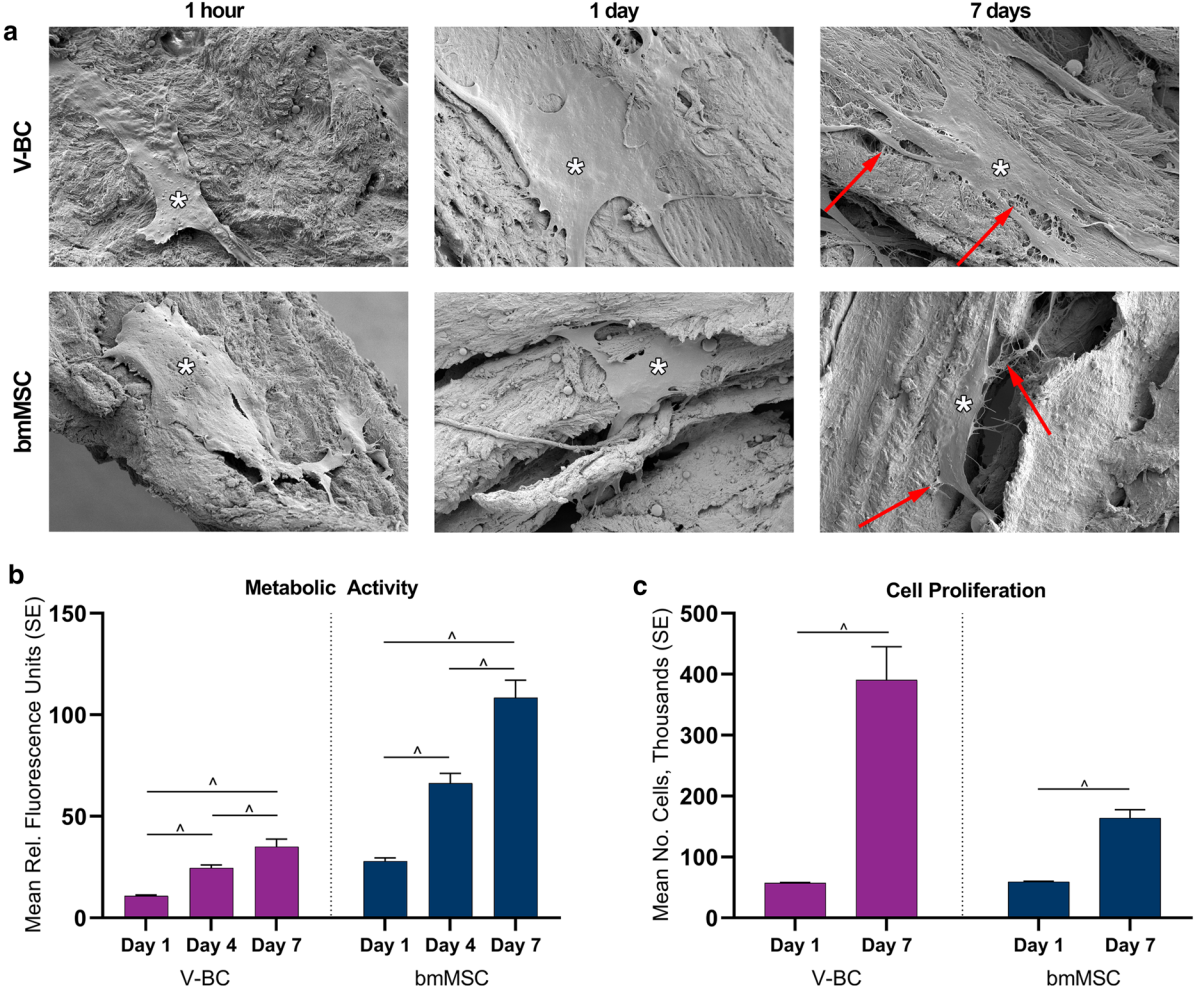
**a** Representative SEM images taken at 1 h, 1 day, or 7 days after V-BC or bmMSC were seeded onto the demineralized component of VBA (3000 × magnification). After 1 h, cells were attached to VBA (*) and demonstrated spreading and extracellular matrix deposition over the course of 7 days, demonstrating the biocompatibility and osteoconductivity of VBA. V-BC and bmMSC seeded onto the demineralized component of VBA showed significantly increased **b** metabolic activity, as assessed by alamarBlue assay, and **c** cell proliferation, as assessed by PicoGreen DNA quantification over the course of 7 days, further indicating the biocompatibility of VBA. ^*p* < 0.05; N = 6

To assess the ability of VBA to provide a biocompatible osteoconductive scaffold for bone remodeling, V-BC or bmMSC were seeded onto VBA, as depicted in Fig. 5a. One hour after cell seeding, V-BC and bmMSC were attached to the demineralized component of VBA. Over the course of 7 days, both cell types spread into cell monolayers and deposited extensive extracellular matrix. Lamellipodia and filopodia were observed extending from the cells and across spaces between the demineralized component.

To further demonstrate the biocompatibility of VBA, the cellular metabolism, assayed by alamarBlue, of V-BC and bmMSC seeded onto the demineralized component of VBA significantly increased over 7 days in culture (Fig. 5b; *p* < 0.05). Further, proliferation and the number of cells, measured by the PicoGreen DNA quantification assay, significantly increased by Day 7 for both cell types (Fig. 5c; *p* < 0.05). Altogether, these results support the biocompatibility of VBA, demonstrating that it provides an osteoconductive scaffold for cellular attachment, spreading, and proliferation.

### *Osteoinductivity: VBA contains relevant growth factors and supports bone formation in vivo*

Bone remodeling requires coordination of both osteoinductive and angiogenic signals (Hankenson et al 2011). Therefore, the presence of relevant growth factors necessary for bone remodeling was assessed in the demineralized component of VBA. ELISA analysis revealed the presence of osteoinductive growth factors, BMP-2 and BMP-7 (Table 1). Additionally, angiogenic growth factors, VEGF and angiogenin, were detected via MAGPIX analysis. Therefore, the demineralized component of VBA provides relevant osteoinductive and angiogenic growth factors for bone remodeling, as reported in the literature (Hankenson et al 2011; Zhang et al 1997).

**Table 1:** Summary of osteoinductive and angiogenic growth factors in VBA

Growth factor^a^, pg/mL	Mean (SE) (N = 6)
BMP-2	1566.02 (279.90)
BMP-7	9016.11 (1503.53)
VEGF	377.49 (74.46)
Angiogenin	285.5 (52.31)

*BMP* bone morphogenetic protein, *SE* standard error of the mean, *VEGF* vascular endothelial growth factor

^a^BMP-2 and -7 were measured via ELISA; VEGF and angiogenin were measured via MAGPIX

**Fig. 6:**
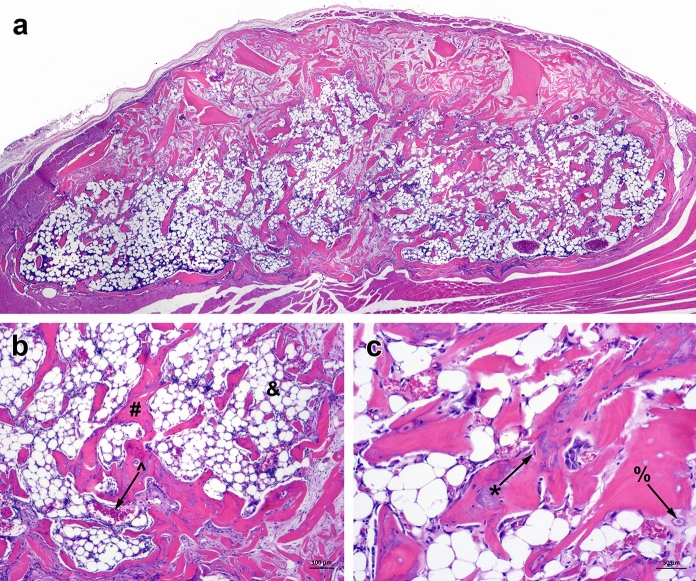
Representative H&E staining of explants from an athymic nude mouse implanted with the demineralized component of VBA. **a** Merged set of images of H&E staining shows more than 50% new bone elements in the entire explant at 35 days post-implantation (4 × magnification). Expanded areas at **b** 4 × and **c** 10 × show the presence of new bone elements including new bone (*), bone marrow (&), new blood vessels (^), and chondrocytes (%) around the implanted demineralized component

In vivo osteoinductive potential was assessed using an athymic nude mouse model, in which the demineralized component of VBA was inter-muscularly implanted for 35 days. Histological evaluation of explants showed more than 50% new bone elements present within and around the implanted bone (Fig. 6a). New bone (*), bone marrow (&), new blood vessels (^), and chondrocytes (%) were observed and depicted in Fig. 6b, c. These data demonstrate the osteoinductive potential of the demineralized component of VBA.

### *Osteogenicity: V-BC produce calcium deposits in vitro*

**Fig. 7:**
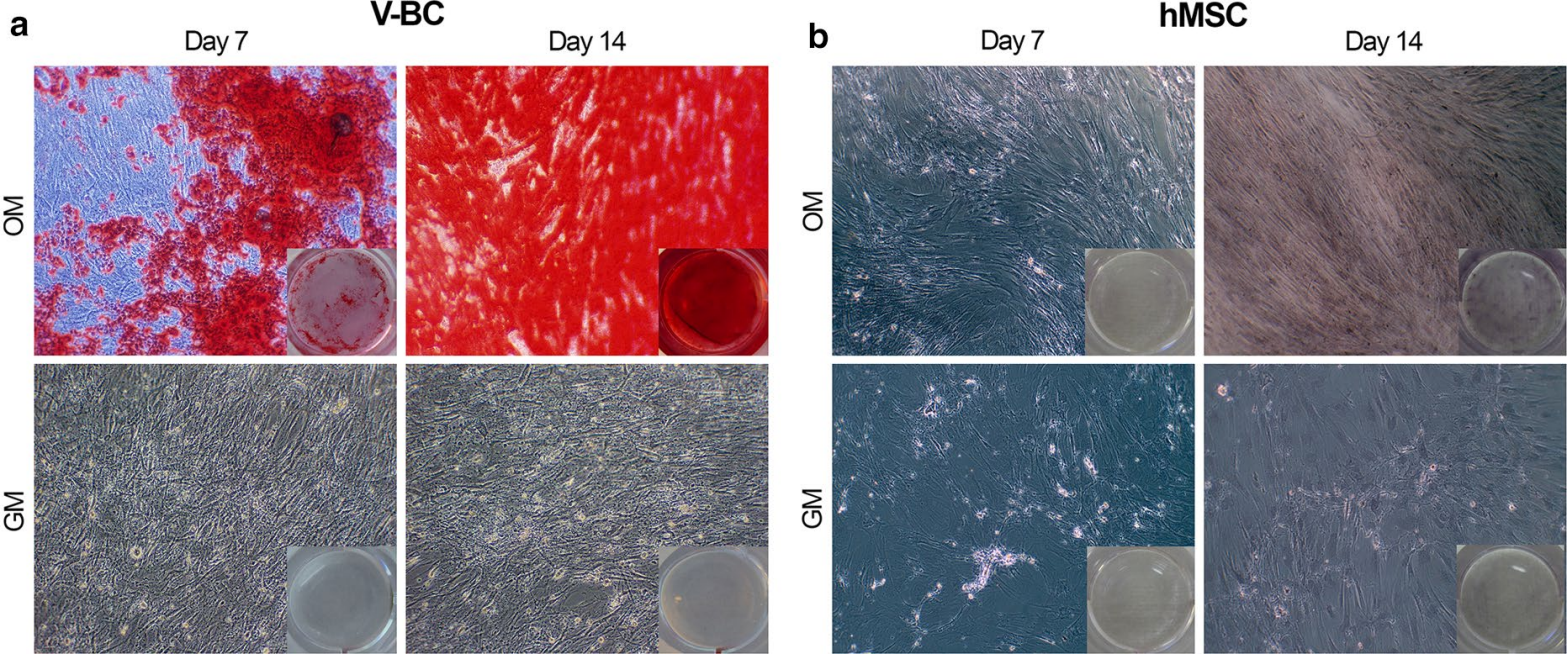
Representative images of Alizarin Red S staining in **a** V-BC or **b** hMSC cultured in either osteogenic media (OM) or growth media (GM) for up to 7 or 14 days (10 × magnification). V-BC demonstrated Alizarin Red S staining at Day 7, which continued up to Day 14 and Day 21 (not shown), demonstrating calcium deposition and osteogenicity. No Alizarin Red S staining was detected from hMSCs at either Day 7 or Day 14. Insets show the gross observation of the entire culture well

To assess the osteogenic potential of VBA, calcium deposition by V-BC over time was evaluated by Alizarin Red S staining (Fig. 7). V-BC cultured in osteogenic induction media demonstrated substantial calcium deposition as early as 7 days in culture, while no calcium was observed when cells were cultured in growth (control) media (Fig. 7a). Calcium deposition increased and was extensively detected after 14 days (Fig. 7a) and up to 21 days in culture (data not shown). In contrast, at the same time points, there was no calcium deposition detected from hMSCs (Fig. 7b), and by Day 21, the cells detached from culture wells (data not shown) with no additional calcium deposition. These results indicate V-BC readily deposit calcium and sooner than hMSCs, which require time to differentiate into bone-forming cells, supporting the osteogenicity of V-BC.

Potential immunogenicity

*VBA is processed to substantially reduce bone marrow cells*

**Fig. 8:**
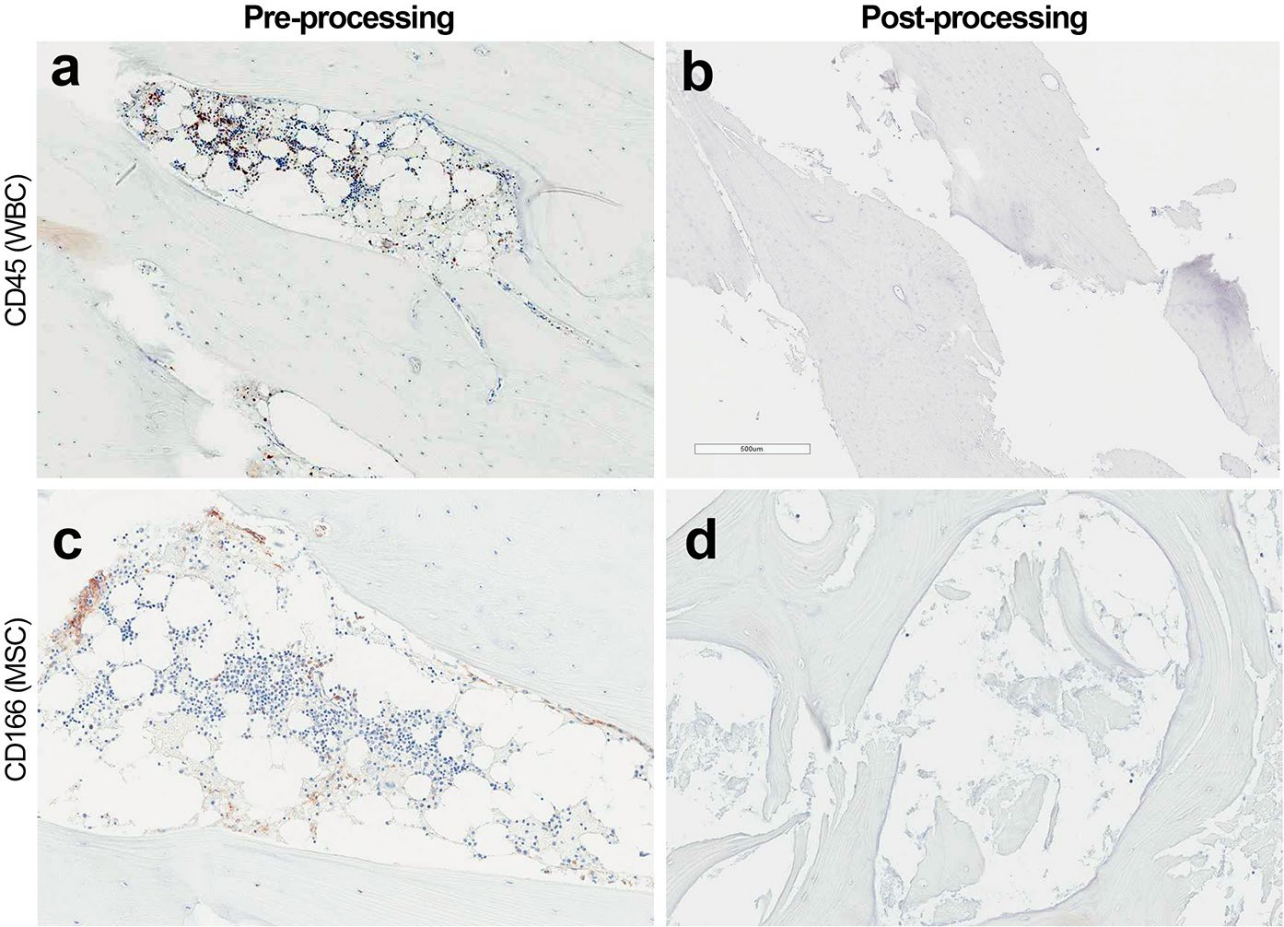
Representative micrographs of IHC staining for bone marrow markers, **a** and **b** CD45 (a marker of hematopoietic cells) or **c** and **d** CD166 (a marker of MSCs) before (*left panels*) and after (*right panels*) proprietary processing, cryopreservation, and thawing of VBA, demonstrating the effective removal of bone marrow components in VBA’s final form (10 × magnification)

The potential immunogenicity of VBA was first evaluated with histological detection of bone marrow components. Representative images of IHC staining of bone matrix samples for CD45, a marker of hematopoietic cells, and CD166, a marker of MSCs, are shown in Fig. 8. Both CD45 and CD166 were detected in the bone marrow of pre-processed bone (Fig. 8a, c) but were absent in the VBA-processed bone (Fig. 8b, d). These results demonstrate the effective removal of bone marrow components in VBA’s final form.

*V-BC did not induce an immune cell response*

**Fig. 9:**
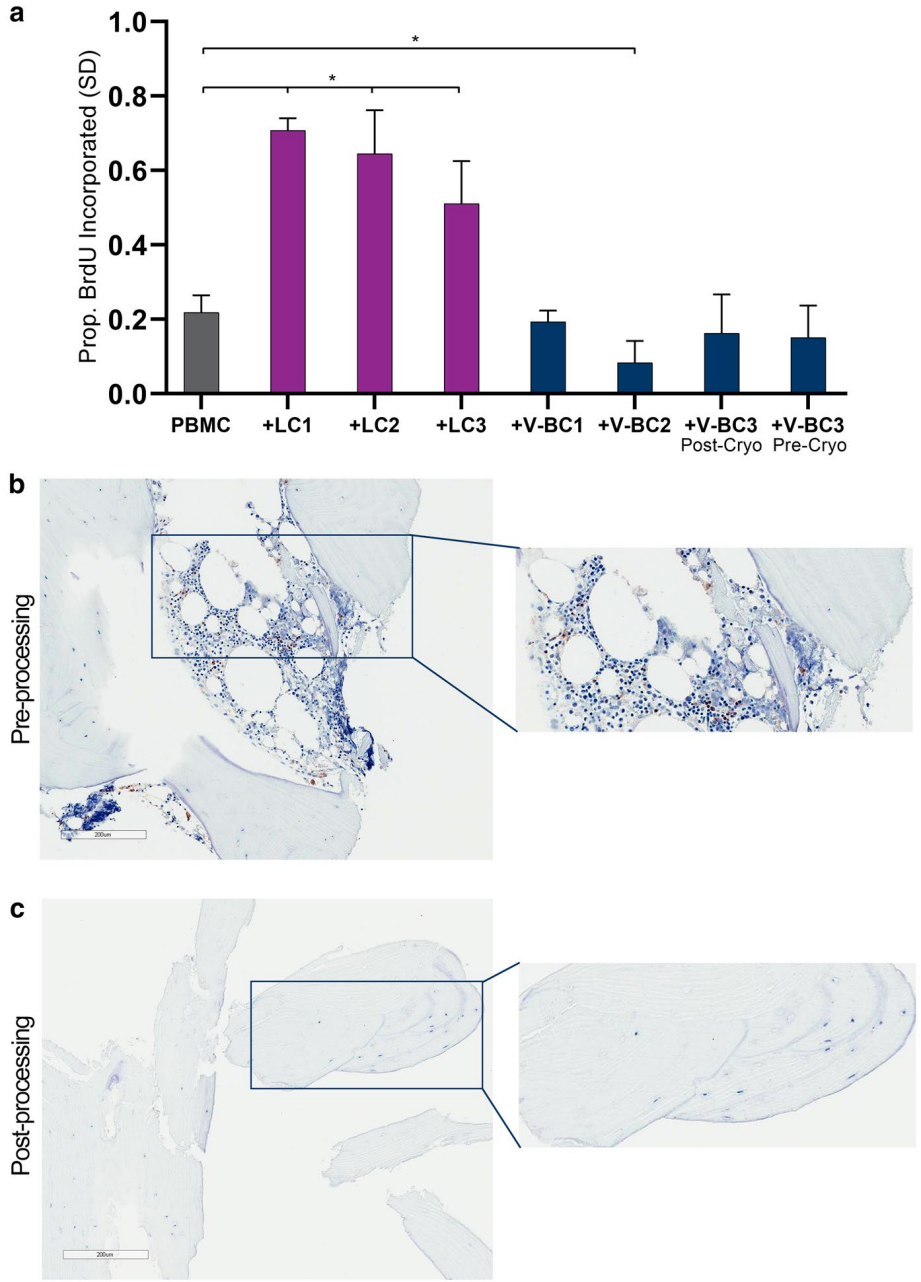
**a** MLR assay results demonstrating that V-BC did not induce an increase in proliferation of the HLA-mismatched PBMCs (as evidenced by no increase in BrdU incorporation compared to the PBMC only control), indicating a lack of immune cell activation. In contrast, LCs derived from the same donors as those for V-BC induced a significant increase in PBMC proliferation, with significantly greater BrdU incorporation into DNA compared to the PBMC only control, indicating immune cell activation. N = 3; *p < 0.05. Lower panels show representative images of bone matrix **b** before and **c** after VBA processing, cryopreservation, and thawing stained for MHCII surface receptors. V-BC within the bone matrix do not stain positive for MHCII, suggesting V-BC are non-immunogenic and providing an explanation of the lack of immune cell activation in the MLR assay. 10 × magnification; insets show magnified area of stained bone matrix

To test the potential immunogenicity of V-BC, a mixed lymphocyte reaction (MLR) assay was performed. V-BC or LCs derived from the same respective donors were co-cultured with a population of HLA-mismatched PBMCs. Co-culture with the LCs induced a significant increase in PBMC proliferation, indicating immune cell activation (*p* < 0.05; Fig. 9a). In contrast, V-BC co-cultured with PBMCs did not induce an increase in proliferation, with the level of proliferation being comparable to that of the untreated PBMC control group. Of note, the PBMC proliferation co-cultured with the V-BC2 sample was significantly lower than the untreated PBMC control. However, this is likely an experimental artifact and not related to the immune cell response. Additionally, a V-BC sample which had not undergone cryopreservation was likewise tested to evaluate the potential effect of cryopreservation on immunogenicity and similarly did not induce an increase in PBMC proliferation. This lack of proliferation indicates that the V-BC did not induce immune cell activation in vitro, suggesting a lack of immunogenicity of cells contained in VBA.

## V-BC did not express MHCII surface receptors

To better understand the lack of immune cell response induced by the V-BC, the presence of the MHCII surface receptors in bone matrix samples was assessed before and after processing. MHCII surface receptors present extracellular antigens to immune cells. However, some cell types have absent, low, or altered conformations of MHCII expression, and as a result, avoid detection by the immune system (Klyushnenkova et al 2005; McIntosh et al 2006). Pre-processed bone showed expression of MHCII in the bone marrow regions, however the cells within the bone matrix did not stain positively for MHCII (Fig. 9b). After VBA processing, the bone marrow was absent and the cells within the bone matrix remained negative for MHCII (Fig. 9c). Therefore, the absence of MHCII expression in the bone cells within VBA further suggests a lack of immunogenic potential, offering an explanation for the non-immunogenicity of V-BC in the MLR assay. This absence of MHCII expression on bone cell surfaces is supported by similar reports in the literature (Khoury and Arnaud 1993; Liu et al 2006).

## Discussion

This paper presents a preclinical characterization of VBA. The results described herein suggest that VBA contains viable cells that are able to proliferate, and that express genes and proteins typically associated with lineage-committed bone cells. Further, the VBA matrix is osteoconductive in that it supports cell attachment, spreading, and proliferation; it contains relevant osteoinductive and angiogenic growth factors and supports bone formation in vivo; and in vitro production of calcium deposits suggests that the viable cells within VBA are osteogenic. Finally, VBA does not contain bone marrow cells and did not elicit an immune response, likely owing to the viable cells within it not expressing MHCII. Taken together, these results suggest that VBA provides all three essential bone remodeling properties and contains viable lineage-committed bone-forming cells, thus providing a potential alternative to autograft without the associated drawbacks.

In clinical cases that are complex or otherwise challenging, the advantages of VBA may be particularly useful. A number of factors in a given case have the potential to adversely affect the success of a bone graft procedure, and therefore, are taken into consideration by surgeons when choosing what type of bone graft to employ. These factors can be patient-related, such as advanced age, comorbidities, and lifestyle risk factors, as well as case-related, including the size and complexity of the defect. Autograft is historically the preferred option; however, limitations in quantity and quality, as well as potential complications associated with a second surgical site (Younger and Chapman 1989) have prompted surgeons to seek alternative graft options. VBA, being more advanced than CBAs, has shown success when used in complex cases and high-risk patients across a range of clinical applications (Alfi et al 2021; Divi and Mikhael 2017; Elgafy et al 2021; Gibson et al 2021; Hall et al 2019; Marschall et al 2019, 2020; Moran et al 2020; Roukis 2018; Roukis and Samsell 2018; Roukis et al 2020; Ryu et al 2021; Shahrdar et al 2020).

For example, in foot and ankle procedures, in which nonunion rates are reported as high as 40% in high-risk patients (Fortin and Beaman 2020), several studies have demonstrated success using VBA in patients with comorbidities (Moran et al 2020; Roukis 2018; Roukis and Samsell 2018; Roukis et al 2020). In particular, Moran, et al (2020) reported an 86% fusion rate in 135 high-risk patients, which was similar to fusion rates reported with the use of autograft (Lareau 2015; Muller 2013). Likewise, in spinal fusion, in addition to patient comorbidities and lifestyle risk factors, the number of levels to be fused can add to the complexity of a case and potentially limit the ability to achieve successful fusion. Hall, et al (2019) reported a 98% successful fusion rate using VBA in instrumented posterior lumbar fusion (IPLF) procedures, in which 59.3% of the patients (89 of 150 patients) had undergone treatment for 4 or more levels. More recently, Elgafy, et al (2021) reported successful fusion in 91.7% of patients undergoing multilevel IPLF and who possessed multiple comorbidities and/or lifestyle risk factors. By comparison, rates of pseudarthrosis in similar procedures using varying graft materials, including autograft or DBM, are reported from 7% to as high as 48% (Cammisa Jr et al. 2004; Lee et al 2009; Sengupta et al 2006). Finally, size of the defect is of considerable concern in bone grafting procedures, with many surgeons adhering to the traditionally held belief that only vascularized grafts should be used in defects larger than 6 cm (Allsopp et al 2016; Foster et al 1999). However, recent reports using VBA in craniomaxillofacial applications demonstrated successful bone healing in defects larger than 6 cm, reported at 6.5 cm in a pediatric patient (Alfi et al 2021) and an average of 7.4 ± 0.8 cm in 2 adult patients (Marschall et al 2020). While results from individual cases cannot be generalized, these results support the use of VBA in large (> 6 cm) defects. Altogether, these studies demonstrate VBA is an effective graft alternative to autograft for complex and high-risk cases, including large defects and multi-level spinal fusion and patients with multiple comorbidities and/or lifestyle risk factors.

Prior to the launch of VBA, MSC-based CBAs were the primary allograft option as an alternative to autograft, potentially providing all three essential properties for bone healing. This would be advantageous for complex and high-risk cases, however, previous preclinical research demonstrates that lineage-committed bone-forming cells are more suitable than the more conventional MSCs in promoting bone formation (Birmingham et al 2012; Ghanaati et al 2011; Tortelli et al 2010), and clinical evidence since its introduction supports the advantage of VBA in this regard. A recent study describing two patients who underwent two-stage total hip arthroplasty with VBA provided a unique opportunity to evaluate the graft histologically after clinical implantation (Shahrdar et al 2020). In one patient, over 100 µm of new bone had formed in and around the VBA bone components after only 7 weeks, suggesting that formation began shortly after implantation. Histology also confirmed neovascularization, supporting the angiogenic potential of VBA. Likewise, these results were consistent in the second patient who possessed a greater number of risk factors (64-year-old, female with Type I diabetes and osteoporosis). Notably, neither patient showed signs of immune rejection. These histological findings support VBA’s ability to readily lay down bone matrix and support neovascularization shortly after clinical implantation, consistent with the preclinical results described in the present study.

While direct clinical studies comparing VBA with traditional MSC-based CBAs are limited, the published results to date suggest improved outcomes with VBA. In one head-to-head comparison of VBA and an MSC-based CBA (Trinity Evolution^®^ and Trinity Elite^®^; MTF Biologics, Edison NJ; Roukis et al 2020) in foot and ankle arthrodesis procedures, the rate of ankle fusion at 6 months was significantly greater in patients who received VBA (100%) than was observed in patients who received Trinity (50%; *p* < 0.0001). VBA patients also reported significantly greater improvement in pain scores and overall satisfaction. Other recent literature also reports a lower rate of fusion in foot and ankle procedures with MSC-based CBAs, from 68 to 79% (Jones et al 2015; Loveland et al 2017). Similarly, reported fusion rates in the spine are generally lower with MSC-based CBAs (68 to 87%; McAnany et al 2016; Overley et al 2019) than those with VBA (92 to 98%; Elgafy et al 2021; Hall et al 2019). Overall, these data demonstrate a clinical advantage from VBA containing lineage-committed bone-forming cells, compared with traditional MSC-based CBAs.

Finally, economic considerations are another important factor in the choice of bone graft, as reduction of cost is an ever-increasing priority in modern healthcare systems (McGrath et al 2021). Along these lines, recombinant human bone morphogenetic protein-2 (rhBMP-2) has been widely used in spinal fusion due to its demonstrated osteoinductive efficacy, as well as early association with lower index and follow-up costs compared with the historically-preferred autologous iliac crest bone graft (ICBG; Glassman et al 2008a, 2008b). However, rhBMP-2 has since become widely associated with increased costs (Alvin et al 2016; Jain et al 2020). To this end, a recent comparison of 16,172 US lumbar fusion surgeries found that patients receiving VBA had $51,130 less in mean hospital charges for the initial procedure than rhBMP-2 patients, and $22,091 less in mean 12-month follow-up hospitalization charges (Wetzell et al 2020). A subsequent report extended these findings to 24 months, demonstrating $25,302 lower overall follow-up hospitalization charges in the same VBA patients versus the rhBMP-2 patients during this period (Wetzell et al 2021). Yet, in both studies, VBA and rhBMP-2 exhibited similar rates of subsequent lumbar fusions and potentially-relevant hospital readmissions at 12 and 24 months (Wetzell et al 2021, 2020). The similarity in subsequent lumbar fusions at 24 months is of particular interest, as reduction in 24-month revisions due to pseudarthrosis has been repeatedly cited as the most cost-effective attribute of rhBMP-2 and a principal justification of the higher costs associated with it (Jain et al 2020; Safaee et al 2019; Zhang et al 2014). Although not a pure measure of pseudarthrosis, such cases would be included in subsequent lumbar fusion procedures, thus suggesting that the clinical performance of VBA in spinal fusion is at least equivalent to that of rhBMP-2. Coupled with the association between VBA and substantially-reduced hospitalization charges at index through 24 months of follow-up versus rhBMP-2, these results suggest that VBA is the more cost-effective option in spinal fusion.

In summary, the preclinical data presented herein, combined with the published clinical and economic studies, demonstrate VBA as an advanced bone graft option, and an appropriate and efficacious autograft alternative. VBA provides all three essential bone remodeling properties and contains lineage-committed bone-forming cells, offering a clinical advantage over MSC-based CBAs and the ability to lay down new bone shortly after implantation. Clinical studies to date show successful fusion rates, comparable to autograft, using VBA across a range of clinical applications and in complex and high-risk cases. Further, recent studies indicate VBA may be a more cost-effective graft option versus rhBMP2, with similar clinical efficacy, suggesting an economic advantage as well. Thus, the biologic, clinical, and economic evidence combined support VBA as an effective autograft alternative, in particular for complex and high-risk cases in which risk of nonunion is a concern.

## Data Availability

The datasets generated during and/or analyzed in this study are available from the corresponding author upon reasonable request.

## References

[CR1] Albee FH (1915) Bone graft surgery. W.B. Saunders Company, Philadelphia

[CR2] Alfi DM, Hassan A, East SM, Gianulis EC (2021). Immediate mandibular reconstruction using a cellular bone allograft following tumor resection in a pediatric patient. Face.

[CR3] Allsopp BJ, Hunter-Smith DJ, Rozen WM (2016). Vascularized versus nonvascularized bone grafts: What is the evidence?. Clin Orthop Relat Res.

[CR4] Alvin MD, Derakhshan A, Lubelski D, Abdullah KG, Whitmore RG, Benzel EC (2016). Cost-utility analysis of 1-and 2-level dorsal lumbar fusions with and without recombinant human bone morphogenic protein-2 at 1-year follow-up. J Spinal Disord Tech.

[CR5] Birmingham E, Niebur G, McHugh PE (2012) Osteogenic differentiation of mesenchymal stem cells is regulated by osteocyte and osteoblast cells in a simplified bone niche. Eur Cells Mater 23:13–27. 10.22203/ecm.v023a02 10.22203/ecm.v023a02 22241610

[CR6] Cammisa Jr FP, Lowery G, Garfin SR, Geisler FH, Klara PM, McGuire RA, et al (2004) Two-year fusion rate equivalency between Grafton® DBM gel and autograft in posterolateral spine fusion: a prospective controlled trial employing a side-by-side Comparison in the Same Patient. Spine (Phila Pa 1976) 29(6):660–666 10.1097/01.brs.0000116588.17129.b9 15014276

[CR7] CBER (1999) 'Immunotoxicity Testing Guidance' May 6 US Department of Health and Human Services: Food and Drug Administration. Available at: https://www.fda.gov/media/72621/download (Accessed: November 2021).

[CR8] de Boer HH (1988). The history of bone grafts. Clin Orthop Relat Res.

[CR9] Divi SN, Mikhael MM (2017). Use of allogenic mesenchymal cellular bone matrix in anterior and posterior cervical spinal fusion: a case series of 21 patients. Asian Spine J.

[CR10] Elgafy H, Wetzell B, Gillette M, Semaan H, Rowland A, Balboa CA (2021). Lumbar spine fusion outcomes using a cellular bone allograft with lineage-committed bone-forming cells in 96 patients *BMC Musculoskelet*. Disord.

[CR11] Fortin PT, Beaman DN (2020) Revision of Nonunion and Malunion: Ankle Arthrodesis. Revision surgery of the foot and ankle (pp. 313–334). Springer. 10.1007/978-3-030-29969-9_19.

[CR12] Foster RD, Anthony JP, Sharma A, Pogrel MA (1999). Vascularized bone flaps versus nonvascularized bone grafts for mandibular reconstruction: an outcome analysis of primary bony union and endosseous implant success. Head Neck J Sci Specialties Head Neck.

[CR13] Ghanaati S, Unger RE, Webber MJ, Barbeck M, Orth C, Kirkpatrick JA (2011). Scaffold vascularization in vivo driven by primary human osteoblasts in concert with host inflammatory cells. Biomaterials.

[CR14] Gibson AW, Feroze AH, Greil ME, McGrath ME, Sivakanthan S, White-Dzuro GA (2021). Cellular allograft for multilevel stand-alone anterior cervical discectomy and fusion. Neurosurg Focus.

[CR15] Glassman SD, Carreon LY, Campbell MJ, Johnson JR, Puno RM, Djurasovic M (2008). The perioperative cost of Infuse bone graft in posterolateral lumbar spine fusion. The Spine Journal.

[CR16] Glassman SD, Carreon LY, Djurasovic M, Campbell MJ, Puno RM, Johnson JR, et al (2008b) RhBMP-2 versus iliac crest bone graft for lumbar spine fusion: a randomized, controlled trial in patients over sixty years of age. Spine (Phila Pa 1976) 33(26):2843–2849. 10.1097/BRS.0b013e318190705d 10.1097/BRS.0b013e318190705d 19092613

[CR17] Greenwald AS, Boden SD, Goldberg VM, Khan Y, Laurencin CT, Rosier RN (2001). Bone-graft substitutes: facts, fictions, and applications. JBJS.

[CR18] Hall JF, McLean JB, Jones SM, Moore MA, Nicholson MD, Dorsch KA (2019). Multilevel instrumented posterolateral lumbar spine fusion with an allogeneic cellular bone graft. J Orthop Surg Res.

[CR19] Hankenson KD, Dishowitz M, Gray C, Schenker M (2011). Angiogenesis in bone regeneration. Injury.

[CR20] Jähn K, Stoddart MJ (2011). Viability assessment of osteocytes using histological lactate dehydrogenase activity staining on human cancellous bone sections. Methods Mol Biol.

[CR21] Jain A, Yeramaneni S, Kebaish KM, Raad M, Gum JL, Klineberg EO, et al (2020) Cost-utility analysis of rhBMP-2 use in adult spinal deformity surgery. Spine (Phila Pa 1976) 45(14):1009–1015. 10.1097/brs.0000000000003442 10.1097/BRS.0000000000003442 32097274

[CR22] James CDT (1974). Sir William Macewen. Proc R Soc Med.

[CR23] Jones CP, Loveland J, Atkinson BL, Ryaby JT, Linovitz RJ, Nunley JA (2015). Prospective, multicenter evaluation of allogeneic bone matrix containing viable osteogenic cells in foot and/or ankle arthrodesis. Foot Ankle Int.

[CR24] Khan SN, Cammisa FP, Sandhu HS, Diwan AD, Girardi FP, Lane JM (2005). The biology of bone grafting. J Am Acad Orthop Surg.

[CR25] Khoury EL, Arnaud CD (1993). Alkaline phosphatase-positive human osteoblasts do not normally express MHC class II antigens in vivo. Bone.

[CR26] Klyushnenkova E, Mosca JD, Zernetkina V, Majumdar MK, Beggs KJ, Simonetti DW (2005). T cell responses to allogeneic human mesenchymal stem cells: immunogenicity, tolerance, and suppression. J Biomed Sci.

[CR27] Lee S-C, Chen J-F, Wu C-T, Lee S-T (2009). In situ local autograft for instrumented lower lumbar or lumbosacral posterolateral fusion. J Clin Neurosci.

[CR28] LifeNet Health® (2019) 'ViviGen® Cellular Bone Matrix: Instruction for Use'. 63–0146. Available at: https://www.lifenethealth.org/sites/default/files/files/63-0146.pdf

[CR29] Liu H, Kemeny DM, Heng BC, Ouyang HW, Melendez AJ, Cao T (2006). The immunogenicity and immunomodulatory function of osteogenic cells differentiated from mesenchymal stem cells. J Immunol.

[CR30] Loveland J, Waldorff E, He D, Atkinson B (2017). A retrospective clinical comparison of two allogeneic bone matrices containing viable osteogenic cells in patients undergoing foot and/or ankle arthrodesis. J Stem Cell Res Therapy.

[CR31] Marschall JS, Dutra V, Flint RL, Kushner GM, Alpert B, Scarfe W, et al (2019). In-house digital workflow for the management of acute mandible fractures. J Oral Maxillofac Surg 77(10):2084 e1–2084 e9. 10.1016/j.joms.2019.05.027 10.1016/j.joms.2019.05.027 31278940

[CR32] Marschall JS, Kushner GM, Flint RL, Jones LC, Alpert B (2020). Immediate reconstruction of segmental mandibular defects with nonvascular bone grafts: a 30-year perspective. J Oral Maxillofac Surg 78(11):2099 e1–2099 e9. 10.1016/j.joms.2020.03.035 10.1016/j.joms.2020.03.035 33131550

[CR33] McAnany SJ, Ahn J, Elboghdady IM, Marquez-Lara A, Ashraf N, Svovrlj B (2016). Mesenchymal stem cell allograft as a fusion adjunct in one- and two-level anterior cervical discectomy and fusion: a matched cohort analysis. Spine J.

[CR34] McGrath M, Feroze AH, Nistal D, Robinson E, Saigal R (2021). Impact of surgeon rhBMP-2 cost awareness on complication rates and health system costs for spinal arthrodesis. Neurosurg Focus.

[CR35] McIntosh K, Zvonic S, Garrett S, Mitchell JB, Floyd ZE, Hammill L (2006). The immunogenicity of human adipose-derived cells: temporal changes in vitro. Stem Cells.

[CR36] Moran T, Sequeira S, Cooper M, Park J (2020) A retrospective analysis of outcomes from foot and ankle arthrodesis and open reduction and internal fixation using cellular bone allograft augmentation. Foot Ankle Specialist. Epub ahead of print. 10.1177/1938640020952301 10.1177/1938640020952301 32865044

[CR37] Muul LM, Heine G, Silvin C, James SP, Candotti F, Radbruch A, et al (2011) Measurement of proliferative responses of cultured lymphocytes. Curr Protoc Immunol 94(1): 1–7. 10.1002/0471142735.im0710s94 10.1002/0471142735.im0710s94 21809319

[CR38] Overley SC, McAnany SJ, Anwar MA, Merrill RK, Lovy A, Guzman JZ, et al (2019). Predictive factors and rates of fusion in minimally invasive transforaminal lumbar interbody fusion utilizing rhBMP-2 or mesenchymal stem cells. Int J Spine Surg 13(1):46–52. 10.14444/6007 10.14444/6007 PMC6383451 30805286

[CR39] Roukis TS (2018) Use of living cellular bone matrix for treating a failed ankle arthroplasty: an abbreviated technique and case study. Clin Res Foot Ankle 06(03). 10.4172/2329-910x.1000282

[CR40] Roukis TS, Samsell B (2018) A new approach to ankle and foot arthrodesis procedures using a living cellular bone matrix: a case series. Clin Res Foot Ankle 06(03). 10.4172/2329-910x.1000274

[CR41] Roukis TS, Wetzell B, McLean JB, Dorsch KA, Moore MA (2020) A retrospective comparison of clinical and patient-reported outcomes in foot and ankle arthrodesis procedures using two cellular bone allografts. Clin Res Foot Ankle 8(4). https://www.omicsonline.org/open-access/a-retrospective-comparison-of-clinical-and-patientreported-outcomes-in-foot-and-ankle-arthrodesis-procedures-using-two-c.pdf.

[CR42] Ryu B, Abraham C, Polido WD (2021) Treatment of mandibular non-union using patient specific crib cage plates and cellular bone allograft: a case report. Craniomaxillofacial Trauma Reconstruction Open 6. 10.1177/24727512211005949

[CR43] Safaee MM, Dalle Ore CL, Zygourakis CC, Deviren V, Ames CP (2019). Estimating a price point for cost-benefit of bone morphogenetic protein in pseudarthrosis prevention for adult spinal deformity surgery. J Neurosurg Spine.

[CR44] Sengupta DK, Truumees E, Patel CK, Kazmierczak C, Hughes B, Elders G, et al (2006). Outcome of local bone versus autogenous iliac crest bone graft in the instrumented posterolateral fusion of the lumbar spine. Spine (Phila Pa 1976), 31(9), 985–991. Doi: 10.1097/01.brs.0000215048.51237.3c 10.1097/01.brs.0000215048.51237.3c 16641774

[CR45] Shahrdar C, McLean J, Gianulis E, Softic D, Qin X, Moore MA (2020). Clinical outcome and explant histology after using a cellular bone allograft in two-stage total hip arthroplasty. J Orthop Surg Res.

[CR46] Tortelli F, Tasso R, Loiacono F, Cancedda R (2010). The development of tissue-engineered bone of different origin through endochondral and intramembranous ossification following the implantation of mesenchymal stem cells and osteoblasts in a murine model. Biomaterials.

[CR47] Turonis JW, McPherson JC, Cuenin MF, Hokett SD, Peacock ME, Sharawy M (2006). The effect of residual calcium in decalcified freeze-dried bone allograft in a critical-sized defect in the Rattus norvegicus calvarium. J Oral Implantol.

[CR48] Vaananen HK, Zhao H, Mulari M, Halleen JM (2000). The cell biology of osteoclast function. J Cell Sci.

[CR49] Wetzell B, McLean JB, Dorsch KA, Moore MA (2021) A 24-month retrospective update: follow-up hospitalization charges and readmissions in us lumbar fusion surgeries using a cellular bone allograft (CBA) versus Recombinant Human Bone Morphogenetic Protein-2 (rhBMP-2). J Orthop Surg Res 16(680). 10.1186/s13018-021-02829-0 10.1186/s13018-021-02829-0 PMC8600873 34794470

[CR50] Wetzell B, McLean JB, Moore MA, Kondragunta V, & Dorsch KA (2020) A large database study of hospitalization charges and follow-up diagnoses in us lumbar fusion surgeries using a cellular bone allograft (CBA) versus recombinant human bone morphogenetic protein-2 (rhBMP-2). J Orthop Surg Res 15(544). 10.1186/s13018-020-02078-7 10.1186/s13018-020-02078-7 PMC7678152 33213484

[CR51] Wong SY, Dunstan CR, Evans RA, Hills E (1982). The determination of bone viability: a histochemical method for identification of lactate dehydrogenase activity in osteocytes in fresh calcified and decalcified sections of human bone. Pathology.

[CR52] Younger EM, Chapman MW (1989). Morbidity at bone graft donor sites. J Orthop Trauma.

[CR53] Zhang H, Wang F, Ding L, Zhang Z, Sun D, Feng X (2014). A meta analysis of lumbar spinal fusion surgery using bone morphogenetic proteins and autologous iliac crest bone graft. PLoS ONE.

[CR54] Zhang M, Powers RM, Wolfinbarger L (1997). Effect(s) of the demineralization process on the osteoinductivity of demineralized bone matrix. J Periodontol.

[CR55] Zoch ML, Clemens TL, Riddle RC (2016). New insights into the biology of osteocalcin. Bone.

